# Bilingualism, social cognition and executive functions: A tale of chickens and eggs

**DOI:** 10.1016/j.neuropsychologia.2016.08.029

**Published:** 2016-10

**Authors:** Simon R. Cox, Thomas H. Bak, Michael Allerhand, Paul Redmond, John M. Starr, Ian J. Deary, Sarah E. MacPherson

**Affiliations:** a Centre for Cognitive Aging and Cognitive Epidemiology, University of Edinburgh, UK; b Department of Psychology, University of Edinburgh, 7 Geroge Square, Edinburgh EH8 9JZ, UK; c Geriatric Medicine, University of Edinburgh, UK; d Alzheimer Scotland Dementia Research Centre, University of Edinburgh, UK

**Keywords:** Bilingualism, Executive functions, Social cognition, Childhood intelligence

## Abstract

The influence of bilingualism on cognitive functioning is currently a topic of intense scientific debate. The strongest evidence for a cognitive benefit of bilingualism has been demonstrated in executive functions. However, the causal direction of the relationship remains unclear: does learning other languages improve executive functions or are people with better executive abilities more likely to become bilingual?

To address this, we examined 90 male participants of the Lothian Birth Cohort 1936; 26 were bilingual, 64 monolingual. All participants underwent an intelligence test at age 11 years and were assessed on a wide range of executive and social cognition tasks at age 74. The only notable differences between both groups were found for the Simon Effect (which indexes stimulus-response conflict resolution; *β*=−.518, *p*=0.025) and a trend effect for the Faux Pas task (a measure of complex theory of mind; ToM, *β*=0.432, *p*=0.060). Controlling for the influence of childhood intelligence, parental and own social class significantly attenuated the bilingual advantage on the Faux Pas test (*β*=0.058, *p*=0.816), whereas the Simon task advantage remained (*β*=−.589, *p*=0.049).

We find some weak evidence that the relationship between bilingualism and cognitive functions may be selective and bi-directional. Pre-existing cognitive and social class differences from childhood may influence both ToM ability in older age and the likelihood of learning another language; yet, bilingualism does not appear to independently contribute to Faux Pas score. Conversely, learning a second language is related to better conflict processing, irrespective of initial childhood ability or social class.

## Highlights

• We assessed executive functions and social cognition in 90 men aged 74 years.
• We found no difference between bilinguals and monolinguals on the majority of tests.
• Bilinguals scored higher than monolinguals on the Faux Pas task and Simon Task.
• Differences on the Faux Pas task were attenuated by age 11 IQ and social class.
• Bilingual advantage on the Simon Task was independent of age 11 IQ and social class.

## 1 Introduction

Whether bilingualism influences cognitive functions beyond language is a subject of intense debate. On one hand, behavioral studies in children (Calvo and Bialystok, 2014; Kapa and Colombo, 2013; Kovács and Mehler, 2009), young adults (Bak et al. 2014a; Vega-Mendoza et al., 2015) and older adults (Bak et al., 2014a, Bak et al., 2014b; Kavé et al., 2008) have reported better performance in bilinguals than monolinguals on certain cognitive tasks, particularly those measuring the ability to ignore conflicting and/or irrelevant information (Bak, 2016a, Bialystok et al., 2004, Costa et al., 2009, Johnson, 1991). Some studies also report differences in visual memory and spatial processing (Kerrigan et al., 2016). Bilinguals have also been reported to develop dementia 4 years later than monolinguals (Alladi et al., 2013, Bialystok et al., 2007, Freedman et al., 2014, Woumans et al., 2015) and to have a better cognitive outcome after stroke (Alladi et al., 2016). The behavioural data are further supported by neuroimaging results, suggesting systematic differences in brain activation between mono- and bilingual subjects (Bialystok et al., 2016).

On the other hand, there are studies involving children (Antón et al., 2014, Duñabeitia et al., 2014, Gathercole et al., 2014), young adults (Paap and Greenberg, 2013) and dementia patients (Yeung et al., 2014, Zahodne et al., 2014), that have not found differences in performance between bilinguals and monolinguals. It has been argued that the evidence supporting the notion of a “bilingual advantage” has been influenced by a publication bias favouring positive results (de Bruin et al., 2015b). An apposite summary of the sceptical view was provided in the title of a recent article: “bilingual advantages in executive functions might either not exist or be restricted to very specific and undetermined circumstances” (Paap et al., 2015).

The act of unconsciously activating two languages is thought to require the selection of the appropriate language and suppression of irrelevant linguistic information which conflicts with the currently activated schema (Costa et al., 2008, Bialystok and Viswanathan, 2009, Green, 2011). Thus, the putative specificity of a bilingual advantage in studies that report significant differences fits intuitively with the theoretical cognitive requirements of bilingualism. It also fits with our understanding of executive functions as heterogeneous and potentially dissociable higher-order control processes (including executive components e.g., Collette et al., 2006, Shallice and Burgess, 1996, Baddeley, 1996 and the regulation of social behavior e.g., Brazzelli et al., 1994, Eslinger and Damasio, 1985 see MacPherson et al. (2015)).

While the debate about the “bilingual advantage” continues, many authors have pointed out that a topic as complex as the interaction between languages and cognitive functions cannot be reduced to a simple “yes” or “no” question (Bak, 2015, Baum and Titone, 2014). Research results can be influenced by a large number of variables, such as the definition of bilingualism, the type of cognitive tasks employed and the populations under study. In terms of the definitions of bilingualism, the previous focus on what was perceived to be a classical case of bilingualism (early, simultaneous acquisition of more than one language), has now been replaced by the insight that “bilinguals differ in ways that matter” (Baum and Titone, 2014, p. 875).

Many recent studies have used a more inclusive definition, based on the ability to communicate rather than a perfect command (Alladi et al., 2013, Alladi et al., 2016). Indeed, an improvement in cognitive functions has been reported after only one week of an intensive language course (Bak et al., 2016). Conversely, inactive bilinguals, who used to be early balanced bilinguals in their childhood but moved on to an exclusive use of only one language in their later life, perform differently from active bilinguals and more like monolinguals (de Bruin et al., 2015a, de Bruin et al., 2016). Moreover, early and late acquisition of another language might have different effects on the cognitive system, with early acquisition favouring switching, late acquisition favouring inhibition tasks (Bak et al., 2014a; Tao et al., 2011). If this is the case, bilingualism research should take into account the interaction between the type of bilingualism and the type of task performed.

Moreover, since most studies compare groups rather than individuals, the question needs to be asked whether the mono- and bilingual populations might differ from each other, not only in their language characteristics but also in other relevant variables (Bak, 2016b). One class of possible confounding variables is that of systematic differences between bi- and monolingual populations, which are difficult to avoid in countries where bilingualism is associated with immigrants and their descendants (Bialystok et al., 2007, Paap and Greenberg, 2013), or where bi- and monolingual participants are recruited from different parts of the same country (Costa et al., 2008, Antón et al., 2014). In such cases, both groups might differ not only in language but also in other influences on cognitive function such as genetics, lifestyle, diet, social structure and education. These types of confounds have been addressed by recent studies, conducted in countries in which knowledge of different languages is not necessarily connected to immigrant status, such as Belgium (Woumans et al., 2015) or India (Bak and Alladi, 2016).

The second type of confound pertains to within-population variability. This problem becomes particularly relevant in studies examining late bilingualism. While early bilingualism is determined to a large extent by parental choice and societal pressures, late bilingualism often reflects the individual's own choice. So why do some people learn other languages and others do not? Here, the crucial issue is that of so-called “reverse causality” or a confusion between cause and consequence (Baum and Titone, 2014): does bilingualism lead to cognitive differences, or do differences in cognitive ability and social class lead some individuals to become bilingual while others not? Such a quandary is similar to the classic causality dilemma: “which came first, the chicken or the egg? ”, and is extremely difficult to resolve, since data about cognitive performance and social class prior to second language acquisition are required to determine the causal direction.

The Lothian Birth Cohort 1936 (LBC1936) offers a rare opportunity to tackle several of the above-mentioned problems. Firstly, it comprises individuals born in the same year, mostly growing up and spending most of their lives in the same region of Scotland and all being native English speakers. Secondly, they underwent a well-validated intelligence test in 1947, aged 11 years, and have been extensively characterized in their seventies (Deary et al., 2007, Deary et al., 2012). Thus, we are able to examine potential effects of bilingualism on cognition, accounting for the confounding variables of early life intelligence and social class. The first study examining the effects of bilingualism in this cohort demonstrated that bilinguals performed better than monolinguals, particularly on tests of reading and general intelligence, when controlling for age 11 IQ (Bak et al., 2014b). However, this study used general composite measures of cognitive performance and so was unable to examine effects of bilingualism on specific cognitive tasks, in particular on different aspects of social and executive functions. This question is particularly pertinent to current controversies surrounding the exact type of tasks in which a “bilingualism effect” can be detected. Although, as discussed above, there is a considerable controversy as to whether cognitive differences between monolinguals and bilinguals exist at all, there is a broad consensus that ***if*** such differences exist, they would be found above all in the area of executive functions (Bak, 2016a, Valian, 2015).

Against this background, the present study examines the performance of a subset of 90 LBC1936 participants who – in addition to a measure of cognitive ability age 11, parental and own social class – provided scores on 6 tests at ~age 74 years. The tests were selected to tap a variety of executive and social/emotional abilities: the Simon Task, D-KEFS Tower Test, Self-Ordered Pointing Task (SOPT), Faux Pas test, Moral Dilemmas and Reversal Learning. Until now, such an extensive assessment of older bilinguals using a battery comprising both executive and social/emotional tests has not been conducted. Testing an effect before and after adjusting for childhood cognitive ability and social class offers the rare opportunity to control for possible reverse causation (i.e., better cognitive scores and bilingualism in older age might be related because both arise from having higher childhood intelligence and/or class, rather than because bilingualism benefits cognitive scores).

We hypothesized that bilingualism would confer a selective advantage upon some, but not all cognitive functions examined in our study. Based on the previous literature, the main candidates for potential differences are The Simon Task and the Faux Pas Test. In the former test (which involves the difference in response times for congruent and incongruent items), a bilingual advantage has been reported in the past (Kroll and Bialystok, 2013); these results have been contested by subsequent studies (Paap and Greenberg, 2013, Paap et al., 2015), although the small sample size and large confounds in some of them (Kirk et al., 2014) need to be taken into account when interpreting their findings (Bak, 2015). In the latter test (which measures the ability to accurately identify and describe when a social Faux Pas has been committed in a series of stories), there have been reports of a bilingual advantage on tests of social cognition and theory of mind in children (ToM; Rubio-Fernández and Glucksberg, 2012) but to the best of our knowledge these processes have not been examined in older participants.

In contrast, we expected to find no differences between mono- and bilinguals on the other four tests. Our previous study involving the LBC1936 (Bak et al., 2014b) found no major differences in the Moray House Test, comprising mainly of reasoning tasks and accordingly, we did not expect to find differences on the Tower Test (a test of planning which involves rearranging wooden disks on a 3-peg board to replicate a pictured end-state). Indeed, a recent study conducted in the Hebrides found no difference on the Tower of London tests between mono- and bilingual older participants (de Bruin et al., 2015a), and a bilingual advantage on tests of planning akin to the Tower Test, and working memory such as the Self-Ordered Pointing Task (SOPT; participants are required to select each item within an array of abstract designs only once, while they change position after each selection) have predominantly been conducted only among young adults and children (Blom et al., 2014, Festman et al., 2010, Yang et al., 2005). In terms of moral dilemmas (the degree to which participants would endorse a series of hypothetical scenarios which variably pit their personal moral boundaries against the benefit for the greater good), there is some recent literature suggesting an influence of bilingualism (Costa et al., 2014), but in this case the difference is not found between mono- and bilinguals, but between bilinguals’ first and second language. The Reversal Learning test requires participants to identify when a previously-rewarding stimulus ceases to become beneficial by switching to an alternative response schema, so may plausibly be sensitive to the superior mental flexibility putatively exhibited in bilingualism (though it reportedly involves distinct frontal regions to the Simon Task; MacPherson et al., 2015). We are unaware of any bilingualism research using Reversal Learning; thus analyses using this test are purely exploratory.

The current study included a subgroup of the participants who took part in the previous bilingualism study based on the LBC1936 (Bak et al., 2014a, Bak et al., 2014b) and the group size was, therefore, almost ten times smaller (90 as opposed to 853). Accordingly, we expected that possible differences between mono- and bilingual groups might be more difficult to detect. However, based on the analysis of the literature, we hypothesized that ***if*** any differences between both groups should occur, we would expect a bilingual advantage on the Simon Task and the Faux Pas task, and not on the other tasks included in the battery. Furthermore, our aim was to determine whether any possible differences between mono- and bilinguals could be explained by differences in social class and childhood intelligence.

## 2 Material and methods

### 2.1 Participants

Between 2004 and 2007, 1091 participants attended the first wave of the LBC1936 study. Three years later, at age ~73 years, they returned for a second wave of testing, which was completed by 866 (418 female) participants. At the conclusion of this wave (in late 2011), participants were invited to participate in a cortisol study (Cox et al., 2015a, Cox et al., 2015b) during which 6 cognitive tests were administered, aged ~74 years. Participants were invited based on the following inclusion criteria: completed Wave 2 within 1.5 years of the cortisol study start, male (to avoid the confound of gender-based endocrine variation), ≥24 on the Mini-Mental State Exam (MMSE; Folstein et al., 1975), <11 on the depression subscale of the Hospital Anxiety and Depression Scale (Zigmond and Snaith, 1983), not taking antidepressant or glucocorticoid medication, and no reported diagnosis of neurodegenerative disorder, stroke or mini-stroke. Of 118 eligible males, 90 (mean age=74.5 years, SD=0.32; MMSE mean=28.54, SD=1.52) consented and were administered the neuropsychological tests (described in Cox et al. (2014), and also below and in Table 1). Written informed consent was obtained from each participant and the study was conducted in compliance with departmental guidelines on participant testing and the Declaration of Helsinki. Ethical approval was gained from NHS Lothian Research Ethics Committee (NREC:07/MRE10/58) and the Philosophy, Psychology and Language Sciences Research Ethics Committee at the University of Edinburgh.

### 2.2 Assessment of bilingualism

All participants were native English speakers. As previously reported (Bak et al., 2014b), each participant completed a questionnaire about whether they had learned any languages other than English (L2), how many, at what age, and how frequently (daily/weekly/monthly/less than monthly/never) they used them in each of 3 situations (conversation/reading/media). Those who reported being able to communicate in L2 were coded as bilingual.

### 2.3 Cognitive tests

Participants provided a measure of cognitive ability in youth (the Moray House Test), and also 6 tests of executive and social cognition at ~74 years (additional information is available in Cox et al., 2014).

#### 2.3.1 Moray House Test (age 11)

At age 11, the participants took the Moray House Test No. 12 – a multi-domain intelligence test of reasoning, word classification and other verbal, spatial and arithmetical items with a 45 min time limit. The total score was concurrently validated against the Terman-Merrill revision of the Binet Scales (SCRE, 1949).

#### 2.3.2 Simon Task (Simon, 1969)

We administered the Simon Task originally reported by di Pellegrino et al. (2007; translated into English) to assess response competition. We asked participants to respond as quickly and accurately as possible to the appearance of a red or green square on a computer screen by pressing the red or green key (positioned on the A and L keys respectively of a QWERTY keyboard). A single square appeared on either the left or right of the screen, making the required response for a red square incongruent if it appears on the right. The main outcome variable was the Simon Effect (mean RT on incongruent trials/mean RT on congruent trials). A lower score indicates a lower cost of responding to incongruent versus congruent stimuli. The calculation of this effect as a proportion (versus using raw reaction time data) affords control for individual differences in simple processing speed, which have been suggested as a possible confounder of bilingual-monolingual differences on Simon Effect performance (Paap et al., 2015).

#### 2.3.3 Tower Test (D-KEFS; Delis et al., 2001)

This is commonly considered a test of reasoning and planning. Each of the 9 problems began with wooden disks on a 3-peg board in a specific configuration. Participants were asked to rearrange them to a pictured end-state in as few moves as possible by moving the disks according to set rules (e.g., only move one disc at a time, a larger disc can never sit on top of a smaller disc). The main outcome variable was Total Achievement Score (/30) in accordance with D-KEFS scoring booklet, where higher scores indicate superior performance.

#### 2.3.4 Self-Ordered Pointing Task (SOPT; Petrides and Milner, 1982)

A test of working memory and monitoring, this computerized task presented participants with a 3×4 grid of 12 abstract designs (MacPherson et al., 2002) on a touchscreen interface (iiyama ProLite T2250MTS 22″ 1920×1080). The participant was required to select each design only once, choosing an item not previously selected. Following each choice, the order of some of the items in the grid was rearranged to ensure participants remember the previously chosen images by their appearance rather than their location. The array was presented 12 times (one run) and the test ended after three runs had been completed. The outcome measure was the number of times a previously chosen item was selected (higher score reflects more errors), and this was averaged across the three runs.

#### 2.3.5 Reversal Learning (Rolls et al., 1994)

This modified version of a previously reported neuroimaging paradigm (Hampton and O’Doherty, 2007) is considered a test of behavioural flexibility. Two fractal images were presented at once, with the aim of determining which selection will make the most money. One image always gave a win of 25p, and the other always a loss of 25p. Once the correct image was identified (indicated by 8 consecutive correct selections), the stimulus-reward contingency was reversed. This pattern continued for 50 trials, allowing a maximum of 5 reversals after the initial contingency had been learned. The main outcome variable was the total number of errors.

#### 2.3.6 Faux Pas test (Stone et al., 1998, Gregory et al., 2002)

This task requires participants to identify whether a protagonist said something awkward, or something they should not have said in 20 short stories (10 containing a faux pas). Once participants had read each story (self-paced), they were asked questions to determine whether they detected and understood whether a faux pas had occurred, including 2 factual control questions at the end of each story to ensure general understanding. All participants exhibited a good factual understanding (M=39.31, SD=1.3 out of a possible 40). Audio-taped responses were marked in accordance with scoring guidelines (http://www2.psy.uq.edu.au/~stone/Faux_Pas_Recog_Test.pdf). The main outcome was the total number of correct responses to questions about the 10 Faux Pas stories (out of a possible 50, excluding the factual control questions).

#### 2.3.7 Moral Dilemmas Task (Greene et al., 2001)

Participants were shown a series of dilemmas and asked (after each one) whether they would endorse a suggested action to resolve the situation (e.g., “Would you push the stranger onto the tracks in order to save the five workmen? ”). The task was presented on a computer (participants responded with a “y” or “n” key press) using 11 previously reported high-conflict dilemmas (Koenigs et al., 2007, Greene et al., 2001). The main outcome variable was the percentage of suggested actions that each participant endorsed (i.e., a higher proportion of endorsements indicates a greater willingness to contravene personal moral cost for wider benefit; or utilitarianism).

### 2.4 Assessment of Socio-Economic Status (SES)

Participants reported the highest occupational position achieved for both themselves, and for their father. This was assigned a social class from I, professional, to V, unskilled (class III being divided into IIIN and IIIM, nonmanual and manual, respectively) according to the 1951 Classification of Occupations (General Register Office, 1956).

### 2.5 Statistical analysis

A sample size of 90 participants gives 80% power to detect a minimal effect size of Cohen's *f*=0.299, with alpha set at 0.05 in a linear regression. For the purposes of group contrast, this could be translated directly into a partial eta squared of 0.082, and that could be read as adding a covariate which explains about 8.2% of the remaining outcome variance. We fitted two multiple linear regression models for each of the six test z-scores using R function ‘lm’. First, simple regressions on the bilingual variable (0=monolingual, 1=bilingual) were performed. The second model included IQ at age 11, the participant's own and parental class. This model tested whether there were any specific effects of bilingualism on cognitive performance, irrespective of potential confounding factors. For example, higher cognitive ability in older age *and* having learnt a second language could both be accounted for by higher cognitive ability in youth and higher class, rather than because bilingualism is beneficial for older age cognition *per se*. In order to quantify any attenuation of effects by covariates, we compared the fit of the models with and without adjustment for each cognitive test.

Finally, we performed sensitivity analysis in order to examine possible confounding effects of pre-age 11 L2 acquisition on the relationship between L2 acquisition and cognitive abilities (by removing those participants and re-running models).

## 3 Results

Twenty-six participants were classified as bilingual, and 64 as monolingual. Descriptive statistics variables are shown in Table 2, and correlations among the cognitive variables and social class are shown in the appendix (Table A1). From the bilingual group, twenty participants (74%) spoke two languages, with the remaining 7 (26%) speaking more than two. In addition, 11 (41%) classified themselves as active users of a second language. Further information on language use is reported in Table A2. Bilingual participants were no different in age (*t* (88)=−1.14, *p*=0.26), or Father's Social Class (*t* (77)=1.34, *p*=0.19) than monolinguals, but showed a significantly higher age 11 IQ score (*t* (80)=−2.67, *p*<0.01), and Own Social Class (*t* (86)=3.29, *p*<0.01).

Table 3 shows the results of linear models testing for effects of bilingualism on each cognitive test in older age (Model 1), and multiple regressions including age 11 IQ and Social Class as covariates (Model 2). Unstandardized betas are reported throughout. Bilinguals had a significantly smaller Simon Effect (*β*=−0.518, *p*=0.025), and also showed a trend towards better performance on the Faux Pas test in Model 1 (*β*=0.432, *p*=0.060). The bivariate analyses found no other significant effects of bilingualism on any other measure of executive and social function. An F-test to compare the variances of cognitive score between monolinguals and bilinguals was non-significant for all tests except for the Faux Pas test, where bilinguals showed significantly lower variance (*F*=3.1, *p<*0.05).

We next examined whether these effects were independent of childhood intelligence and class, which may possibly confound interpretation of putative bivariate relationships. The multiple linear regression models (Model 2, Table 3) show that, when accounting for these covariates (age 11 IQ and social class), bilinguals did not perform significantly better than monolinguals on the Faux Pas test (*β*=0.058, *p*=0.816). Comparison of the Models 1 and 2 for the Faux Pas tests showed a significant likelihood ratio (*p*<0.001), confirming significant attenuation. The results also indicated that the bilingual effect on Faux Pas score was predominantly attenuated by age 11 IQ. In contrast, the relationship between a smaller Simon Effect and being bilingual remained after accounting for both age 11 IQ and Class (*β*=−0.589, *p*=0.049; non-significant attenuation *p*=0.869). Effects of bilingualism on the Tower Test, Self-Ordered Pointing Task, Dilemmas or Reversal Learning remained non-significant.

Only three participants reported L2 acquisition before age 11. Re-running the analyses excluding these participants did not significantly alter our results (data not shown), suggesting the reported effect is not confounded by L2 learning before the measure of IQ at age 11. In an additional post-hoc analysis, we investigated whether the bilingual advantage on the Simon Task was specific to the Simon Effect, or was also present for accuracy. We found no significant differences between mono- and bilinguals on accuracy either before (*t*(87)=0.189, *p*=0.850) or after (*t*(79)=0.603, *p*=0.550) adjusting for age 11 IQ and social class.

## 4 Discussion

Our results suggest that bilingualism influences executive and social functions, but the way in which it does so is subtle and selective. As expected on the basis of previous studies (Bak et al., 2014b; de Bruin et al., 2015a, de Bruin et al., 2015b), bilinguals and monolinguals showed a similar level of performance on most tests applied in this study, namely Tower Test, Self-Ordered Pointing task and Reversal Learning, indicating that the mono- and bilingual groups in our study are broadly matched on these aspects of executive performance. We also did not find any difference on the Moral Dilemma Task: performing the task in one's non-native language is associated with an altered pattern of preferences (Costa et al., 2014) but in our group, all participants performed the test in their native language.

In line with our hypothesis, the two tasks where the mono- and bilingual groups appeared to perform differently were the Simon Task and the Faux Pas Test (although the latter trend did not reach significance). However, when we took account of childhood intelligence and class, the Faux Pas score was significantly attenuated (predominantly by age 11 IQ). This could indicate that children with better social cognition are more likely to learn a second language and tend to grow up to perform better on tests of complex ToM; bilingualism does not appear to independently benefit ToM abilities, measured by this test. These results are in line with recent findings in children suggesting that the effects of bilingualism on executive functions and on social cognition are dissociable and can be related to different aspects of bilingual experience (Fan et al., 2016).

In contrast, we found that – irrespective of childhood intelligence or social class (which could reflect differences in factors such as access to second language education, for example) – bilinguals appear to have a Simon Effect which is ~0.5 SD lower than monolinguals. This denotes a reduced cost of reaction times when responding to incongruent (compared to congruent) stimuli. The act of unconsciously activating two languages is thought to require the selection of the appropriate language and suppression of irrelevant linguistic information which conflicts with the currently activated schema (Costa et al., 2008, Bialystok and Viswanathan, 2009, Green, 2011). It might be relevant to stress in this context that our participants were not the classical early simultaneous bilinguals, but acquired their additional languages later in their life. Although early acquisition of a language is more likely to lead to its perfect, “native-like” command, it might well be that late acquisition offers more cognitive advantages (Duñabeitia and Carreiras, 2015). In particular, it has been suggested that late acquisition of a new language requires stronger inhibition mechanisms than an early simultaneous one (Bak et al., 2014a; Tao et al., 2011) – an effect, which could have contributed to a better performance on the Simon Task. This effect seems to have persisted, although the majority of our bilingual participants did not use any languages other than English in their everyday life. Although recent research suggest an important role for language practice (Bak et al., 2016, de Bruin et al., 2015a, de Bruin et al., 2016, de Bruin et al., 2015b), there is also ample evidence for automatic (and often unconscious) activation of both languages in bilingual subjects (Thierry and Wu, 2007) and previous studies have documented bilingual effects also in non-active bilinguals (Bak et al., 2014b). Future studies will need to disentangle age of acquisition, proficiency and language use: three important variables, which could influence cognitive functions independently of each other.

Overall, our results are consistent with the hypothesis that bilingualism specifically benefits the relative cost of resolving cognitive conflict (Kroll and Bialystok, 2013), and this effect is independent of individual differences in raw reaction times (see Section 2.3.2), which had been proposed as a possible confound of a bilingual advantage on the Simon Effect (Paap et al., 2015). We note, however, that although our predictions for these specific effects were hypothesis-driven and the effect sizes were moderate, the bilingual effect on the Faux Pas test did not reach significance, and the analysis for the Simon Effect would not survive correction for multiple testing. In spite of the fact that attenuation of effects can be present when the main effect is not significant (i.e. the appropriateness of examining attenuation of an initial effect should be framed in terms of zero versus non-zero, rather than in terms of statistical significance; Hayes, 2009), it is important to exercise interpretative caution for this reason, and due to several other study limitations which we outline below.

Our sample was relatively small with unequal groups (due to the observational design), and because most had learnt L2 after age 11, we lacked power to examine whether the time of L2 acquisition was significant. Acquisition was ascertained via self-report questionnaire rather than an objective test of proficiency; thus we could not test whether bilingualism effects on these cognitive measures was a function of L2 ability. However, recent studies comparing self-reported and objectively measured language proficiency found remarkably high correlation between both variables (Vega-Mendoza et al., 2015). The sample was all male, all born in the same year and lived in the Lothian area of Scotland, which hinders generalizability to other populations. Nevertheless, these can be seen as strengths in that they eliminate important confounds of sex, age and genetic/cultural background. Most participants learned a second language after age 11, so our results are not applicable to the cases of bilingualism in which both languages are acquired in early childhood (as is the case in much of the previous literature). However, the late-acquisition group is of interest in itself and its cognitive profile only begins to be explored (Bak et al., 2014a; Tao et al., 2011).

No study can eliminate all the confounding variables associated with bilingualism (Bak, 2016b). We cannot exclude that the mono- and bilingual could have differed on some lifestyle variables after the acquisition of their second language. Indeed, there might exist some variables influencing cognitive performance, which have not yet been identified. However, in such a case, we would have expected a more general cognitive superiority in the bilingual group rather than the highly circumscribed pattern we have found (the only two tasks on which we found any suggestion of a possible difference were, exactly as predicted, the Simon Task and the Faux Pas task). The debate about the interaction between bilingualism and cognition remains wide open. Our study adds to it one more layer of complexity: childhood intelligence might influence differentially not only specific cognitive functions but also their interaction with bilingualism.

## Conflict of interest

All authors report no conflict of interests.

## Figures and Tables

**Fig. A1:**
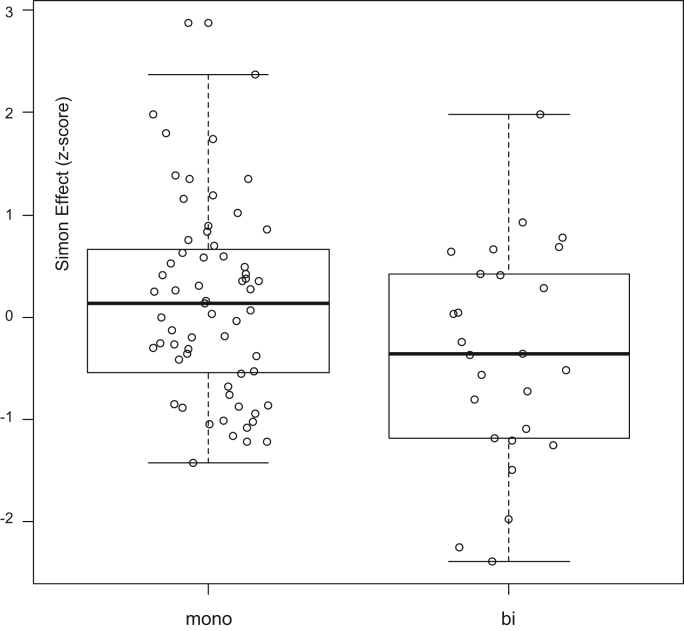
Simon Effect mean and individual differences among monolinguals and bilinguals.

**Table A1:** Correlations among study variables.

		**1**	**2**	**3**	**4**	**5**	**6**	**7**	**8**
**1**	**Age 11IQ**	–							
**2**	**Social Class (O)**	−.549^c^	–						
**3**	**Social Class (F)**	−.108	0.274^b^	–					
**4**	**ToL**	0.350^c^	−.118	−.101	–				
**5**	**Faux Pas**	0.588^c^	−.400^c^	−.190^a^	0.306^c^	–			
**6**	**SOPT**	−.443^c^	0.360^c^	0.056	−.333^c^	−.436^c^	–		
**7**	**Dilemmas**	−.186^a^	0.134	−.048	−.056	−.102	−.017	–	
**8**	**Rev Learn**	−.266^b^	0.377^c^	−.018	−.263^b^	−.253^b^	0.225^b^	0.041	–
**9**	**Simon Effect**	−.197^a^	0.131	−.008	−.213^b^	−.022	−.065	0.193^a^	0.108

Note. Pearson's *r* reported. Social Class (O=Own, F=Father's); ToL=Tower of London; SOPT=Self-Ordered Pointing Task; Rev Learn=Reversal Learning.

a *p*<0.10.

b *p*<0.05.

c *p*<0.001.

**Table A2:** Further language use information.

**Languages Learned**	**Count**
French	15
French & German	1
French & Greek	1
French & Spanish	2
French, Spanish & Arabic	1
German	2
German & French	1
Spanish	3
	

**Languages learned while living abroad**	
French	2
German	1
Spanish	1
Swedish	1

Note. Languages learned: in response to the question: which languages have you learned that you can communicate in? Languages learned while abroad if the participant reported they had spend >1 year overseas and had learnt a local language conversationally.

**Table A3:** R^2^ values for main regression models as shown in Table 3.

**Test**	**Model 1 R**^**2**^	**Model 2 R**^**2**^
Tower	0.019	0.162
Faux Pas	0.041	0.305
SOPT	0.019	0.174
Dilemmas	0.008	0.047
Reversal Learning	0.015	0.167
Simon Task	0.051	0.117

**Table 1:** Summary of cognitive tests.

**Cognitive test**	**Reference**	**Cognitive processes**	**Outcome measure**	
Tower	Delis et al. (2001)	reasoning, planning	Total achievement score	
Faux Pas	Stone et al. (1998)	theory of mind	Total correct responses	
SOPT	Petrides and Milner (1982)	working memory, monitoring	Average number of errors	
Moral Dilemmas	Greene et al. (2001)	moral decision-making	% dilemmas endorsed	
Reversal learning	Rolls et al. (1994)	behavioural flexibility	Total number of errors	
Simon Task	Simon (1969)	stimulus-response conflict processing	Mean incongruent RT	
			Mean congruent RT	

RT=reaction time, SOPT=Self-Ordered Pointing Task.

**Table 2:** Descriptive statistics.

	**Monolingual**	**Bilingual**
**N**	64	26
**Age (years)**	74.45 (0.32)	74.54 (0.31)
**Age 11 IQ**	96.69 (15.95)	107.00 (13.51)
**Own Class**	2.79 (1.02)	2.00 (0.98)
**Father's Class**	3.02 (0.94)	2.68 (1.11)
**Tower of London**	17.22 (3.96)	18.48 (3.86)
**Faux Pas**	38.29 (8.53)	41.63 (4.81)
**Self-Ordered Pointing Task**	2.65 (0.97)	2.36 (0.84)
**Dilemmas**	0.59 (0.23)	0.55 (0.24)
**Reversal Learning**	13.98 (8.41)	11.73 (7.39)
**Simon Effect**	1.09 (0.05)	1.06 (0.06)

Note. Measures given are Mean (SD).

**Table 3:** Regression models of bilingualism on neuropsychological performance.

**Test**	**Variable**	**Model 1**			**Model 2**		
		***β***	**SE**	***p***	***β***	**SE**	***p***
Tower	Bilingual	0.319	0.229	0.167	0.281	0.280	0.320
	age11 IQ				0.020	0.010	0.056
	Social Class (O)				0.256	0.0140	0.071
	Social Class (F)				−0.133	0.123	0.284

Faux Pas	Bilingual	0.432	0.227	0.060	0.058	0.247	0.816
	age11 IQ				0.037	0.009	<0.001
	Social Class (O)				−0.020	0.123	0.872
	Social Class (F)				−0.106	0.108	0.333

SOPT	Bilingual	−0.312	0.230	0.179	−0.086	0.282	0.762
	age11 IQ				−0.024	0.010	0.021
	Social Class (O)				0.095	0.141	0.500
	Social Class (F)				−0.019	0.123	0.877

Dilemmas	Bilingual	−0.175	0.238	0.464	−0.040	0.320	0.900
	age11 IQ				−0.009	0.011	0.444
	Social Class (O)				−0.069	0.161	0.672
	Social Class (F)				−0.082	0.134	0.546

R. L.	Bilingual	−0.276	0.233	0.239	−0.064	0.282	0.822
	age11 IQ				−0.005	0.010	0.636
	Social Class (O)				0.293	0.141	0.041
	Social Class (F)				−0.072	0.122	0.554

Simon	Bilingual	−0.518	0.228	0.025	−0.589	0.293	0.049
	age11 IQ				−0.004	0.010	0.707
	Class (O)				<0.001	0.147	0.999
	Class (F)				−0.052	0.127	0.680

Note. Unstandardized betas are reported. Model 1 shows the effect of bilingualism on each cognitive test score. Model 2 additionally controls for age 11 IQ, and social class. SOPT: Self Ordered Pointing Task; R.L.: Reversal Learning; Simon: Simon Effect; Social Class (O=Own, F=Father's). R^2^ values are shown in Table A3.

## Appendix A

See Fig. A1 and Table A1, Table A2, Table A3.
